# Sterol O-Acyl Transferase 1 as a Prognostic Marker of Adrenocortical Carcinoma

**DOI:** 10.3390/cancers12010247

**Published:** 2020-01-19

**Authors:** Amanda Meneses Ferreira Lacombe, Iberê Cauduro Soares, Beatriz Marinho de Paula Mariani, Mirian Yumie Nishi, João Evangelista Bezerra-Neto, Helaine da Silva Charchar, Vania Balderrama Brondani, Fabio Tanno, Victor Srougi, José Luiz Chambo, Ricardo Miguel Costa de Freitas, Berenice Bilharinho Mendonca, Ana O. Hoff, Madson Q. Almeida, Isabel Weigand, Matthias Kroiss, Maria Claudia Nogueira Zerbini, Maria Candida Barisson Villares Fragoso

**Affiliations:** 1 Unidade de Suprarrenal, Laboratório de Hormônios e Genética Molecular LIM/42, Serviço de Endocrinologia e Metabologia, Hospital das Clínicas, Faculdade de Medicina da Universidade de São Paulo, Av. Dr. Enéas de Carvalho Aguiar, 155, São Paulo, SP 05403-900, Brazil; 2 Serviço de Anatomia Patológica, Instituto do Câncer do Estado de São Paulo (ICESP), Faculdade de Medicina da Universidade de São Paulo, Av. Dr. Arnaldo, 251, São Paulo, SP 01246-000, Brazil; 3 Serviço de Oncologia Clínica, Instituto do Câncer do Estado de São Paulo (ICESP), Faculdade de Medicina da Universidade de São Paulo, Av. Dr. Arnaldo, 251, São Paulo, SP 01246-000, Brazil; 4 Serviço de Urologia, Hospital das Clínicas, Faculdade de Medicina da Universidade de São Paulo, Av. Dr Enéas de Carvalho Aguiar, 255, São Paulo, SP 05403-000, Brazil; 5 Serviço de Radiologia, Instituto do Câncer do Estado de São Paulo (ICESP), Faculdade de Medicina da Universidade de São Paulo, Av. Dr. Arnaldo, 251, São Paulo, SP 01246-000, Brazil; 6 Serviço de Endocrinologia, Instituto do Câncer do Estado de São Paulo (ICESP), Faculdade de Medicina da Universidade de São Paulo, Av. Dr. Arnaldo, 251, São Paulo, SP 01246-000, Brazil; 7 Departamento de Medicina Interna, Unidade de Endocrinologia e Diabetes, Hospital da Universidade de Würzburg, Josef-Schneider-Straße, 97080 Würzburg, Germany; 8 Departamento de Anatomia Patológica, Faculdade de Medicina da Universidade de São Paulo, Av. Dr Arnaldo, 455, São Paulo, SP 01246-903, Brazil

**Keywords:** adrenocortical carcinoma, prognostic factors, SOAT1, target therapies

## Abstract

Adrenocortical carcinoma (ACC) is a rare endocrine malignancy with an unfavorable prognosis. Despite the poor prognosis in the majority of patients, no improvements in treatment strategies have been achieved. Therefore, the discovery of new prognostic biomarkers is of enormous interest. Sterol-O-acyl transferase 1 (SOAT1) is involved in cholesterol esterification and lipid droplet formation. Recently, it was demonstrated that SOAT1 inhibition leads to impaired steroidogenesis and cell viability in ACC. To date, no studies have addressed the impact of SOAT1 expression on ACC prognosis and clinical outcomes. We evaluated SOAT1 expression by quantitative real-time polymerase chain reaction and immunohistochemistry in a tissue microarray of 112 ACCs (Weiss score ≥ 3) from adults treated in a single tertiary center in Brazil. Two independent pathologists evaluated the immunohistochemistry results through a semiquantitative approach (0–4). We aimed to evaluate the correlation between SOAT1 expression and clinical, biochemical and anatomopathological parameters, recurrence-free survival (RFS), progression-free survival (PFS), and overall survival (OS). SOAT1 protein expression was heterogeneous in this cohort, 37.5% of the ACCs demonstrated a strong SOAT1 protein expression (score > 2), while 62.5% demonstrated a weak or absent protein expression (score ≤ 2). Strong SOAT1 protein expression correlated with features of high aggressiveness in ACC, such as excessive tumor cortisol secretion (*p* = 0.01), an advanced disease stage [European Network for the Study of Adrenal Tumors (ENSAT) staging system 3 and 4 (*p* = 0.011)] and a high Ki67 index (*p* = 0.002). In multivariate analysis, strong SOAT1 protein expression was an independent predictor of a reduced OS (hazard ratio (HR) 2.15, confidence interval (CI) 95% 1.26–3.66; *p* = 0.005) in all patients (*n* = 112), and a reduced RFS (HR 2.1, CI 95% 1.09–4.06; *p* = 0.027) in patients with localized disease at diagnosis (*n* = 83). Our findings demonstrated that SOAT1 protein expression has prognostic value in ACC and reinforced the importance of investigating SOAT1 as a possible therapeutic target for patients with ACC.

## 1. Introduction

Adrenocortical carcinoma (ACC) is a rare neoplasia with an estimated incidence of 0.5–2.0/million/year in adults [1,2]. A significant number of patients already present with metastatic disease at diagnosis and have a median overall survival (OS) of 12–15 months and limited therapeutic options. Even after complete resection in cases of localized disease at diagnosis, ACC harbors a significant risk for recurrence and metastatic spread [2,3]; however, this risk is not easily predictable [4]. The exploration of new prognostic biomarkers with possible implications for better decision-making about how to use the available therapies and the development of new therapeutic alternatives is therefore of great interest.

Sterol-O-acyl transferase 1 (*SOAT1*) is a target of steroidogenic factor-1 (SF-1, NR5A1) in the human adrenal [5] and encodes SOAT1 protein. SOAT1 is ubiquitously expressed in most cell types and tissues, but its highest expression is observed in adrenocortical cells (www.proteinatlas.org). SOAT1 is an enzyme present in sub-domains of the endoplasmic reticulum (ER) and catalyzes the formation of cholesterol esters from free cholesterol into lipid droplets (LDs) [6]. This protein is essential for providing intracellular cholesterol homeostasis, maintaining appropriate concentrations of unesterified cholesterol within cells for membrane stability, and protecting adrenal cells from the potentially harmful effects of excess free cholesterol [7].

Emerging evidence demonstrates that lipid metabolism undergoes reprogramming in cancer cells. In glioblastoma (GBM), cholesterol esterification and lipid droplet formation are a hallmark of the neoplasia, correlate with its aggressive behavior, and inversely correlate with OS [8]. In hepatocellular carcinoma, a subgroup of patients characterized by disrupted cholesterol homeostasis present the lowest overall rate of survival and the greatest risk for a poor prognosis; interestingly, the signature of this subgroup is also high SOAT1 expression [9]. In addition, the Human Pathology Atlas data (www.proteinatlas.org/pathology) show that the upregulation of SOAT1 is also significantly correlated with OS in thyroid cancer (*n* = 501, *p* = 0.047), head and neck cancer (*n* = 499, *p* = 0.002), stomach cancer (*n* = 354, *p* = 0.005), and renal cancer (*n* = 877, *p* = 0.007) [10]. Consistently, high levels of SOAT1 expression have also previously been reported to be associated with a poor prognosis in prostate and pancreatic cancer [11,12]. Taken together, these results strongly suggest that the elevated expression of SOAT1 may be a general feature of diverse cancers, and that this protein might be widely used as a prognosis biomarker and therapeutic target for multiple tumors.

In 2015, Sbiera et al. demonstrated that in vitro SOAT1 inhibition led to impaired steroidogenesis and cell viability in ACC, mostly due to ER stress triggered by a reduction in cholesterol esters and an increase in free cholesterol and fatty acids in the intracellular environment. The perpetuation of ER stress led to an increased expression of proapoptotic genes and a reduction in antiapoptotic genes, resulting in cellular apoptosis. In addition, this process resulted in the reduced expression of sterol-responsive genes and, consequently, in reduced steroidogenesis. This same study described SOAT1 as a prominent molecular target for mitotane, the most widely used drug for ACC [6].

To date, no studies have addressed the impact of SOAT1 expression on ACC prognosis and clinical outcomes. The aims of our study were to investigate the expression of SOAT1 at the messenger and protein levels in a large cohort of ACCs in adults and to evaluate the correlation between SOAT1 expression and clinical, biochemical and anatomopathological parameters, recurrence-free survival (RFS), progression-free survival (PFS), and OS.

## 2. Results

### 2.1. SOAT1 Protein Expression

**Figure 1:**
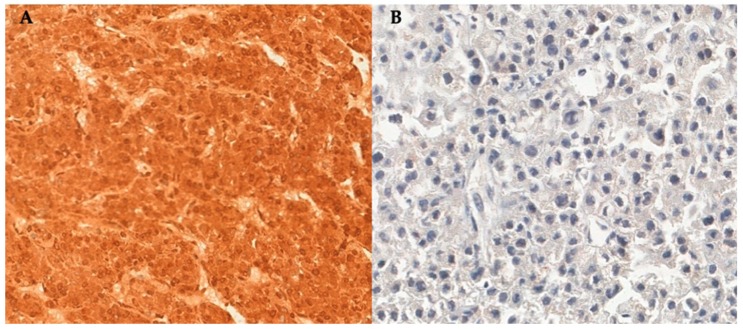
(**A**) Strong immunoreactivity (score 4) for SOAT1 in a cortisol-producing metastatic adrenocortical carcinoma (ACC) in a 30-year-old man presenting an unfavorable outcome with an overall survival of 16 months (400×). (**B**) Absent immunoreactivity (score 0) for SOAT1 in a non-functioning ACC in a 61-year-old woman presenting a favorable outcome after 42 months of follow-up (400×).

Significant heterogeneity in SOAT1 protein expression was observed in our cohort. Strong SOAT1 expression was found in 42 out of 112 carcinomas (37.5%), and a weak or absent SOAT1 protein expression was observed in the remaining cases (Table 1 and Figure 1). Strong SOAT1 protein expression was significantly more frequent in cortisol-producing ACCs, in patients with more advanced disease stage at diagnosis (according to the European Network for the Study of Adrenal Tumors (ENSAT) staging system), and in carcinomas exhibiting a higher Ki67 index (Table 2).

### 2.2. Survival Analysis

**Figure 2:**
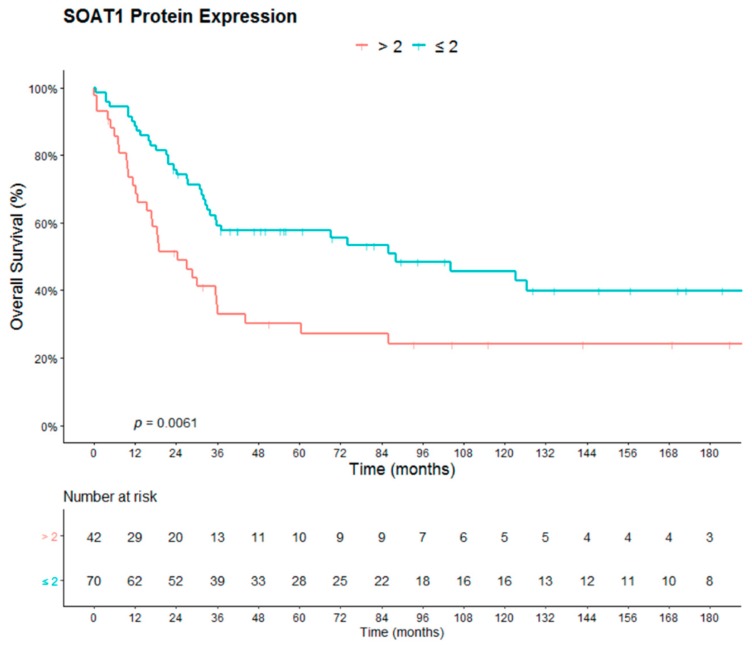
Impact of SOAT1 protein expression on overall survival in 112 adult patients with ACC (tumor samples derived from primary surgery).

**Figure 3:**
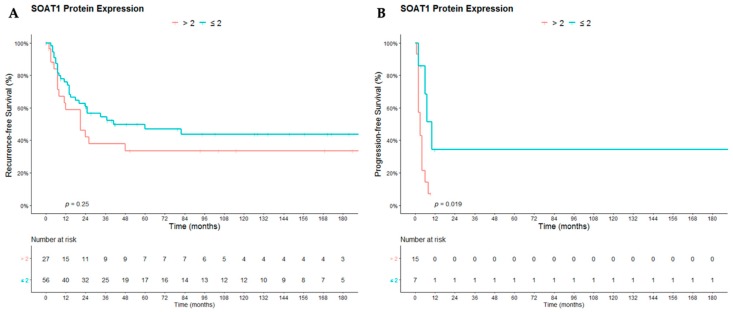
Impact of SOAT1 protein expression on (**A**) recurrence-free survival and (**B**) progression-free survival in adult patients with ACC. For recurrence-free survival analysis, only patients with localized disease at diagnosis who underwent complete resection of the primary tumor (status of resection R0) have been analyzed, and for progression-free survival, only metastatic patients at diagnosis have been analyzed.

Strong SOAT1 protein expression was significantly correlated with reduced OS (*p* = 0.0061) in ACCs (Figure 2). The 112 cases were separated into two different groups according to ENSAT stage at diagnosis, and we evaluated the impact of SOAT1 protein expression on PFS in the subgroup of patients with metastatic disease at diagnosis (ENSAT 4; *n* = 22), and on RFS in the subgroup of patients with localized disease at diagnosis who underwent complete surgical resection of the primary tumor (resection status R0 (*n* = 83)). In addition, strong SOAT1 protein expression solely correlated with a reduced PFS (*p* = 0.019) but did not correlate with a reduced RFS (*p* = 0.25 (Figure 3)).

Regarding OS, Cushing’s syndrome (*p* = 0.013), an ENSAT 3/4 stage (*p* < 0.0001), a Weiss score > 6 (*p* < 0.0001), a Ki67 index > 10% (*p* < 0.0001), and strong SOAT1 protein expression (*p* = 0.007), were associated with reduced OS. In multivariate analysis, only an ENSAT 3/4 stage (hazard ratio (HR) 2.93, 95% confidence interval (CI) 1.68–5.13; *p* < 0.0001), a Ki67 index > 10% (HR 3.11, 95% CI 1.71–5.67; *p* < 0.0001), and strong SOAT1 protein expression (HR 2.15, 95% CI 1.26–3.66; *p* = 0.005) remained predictors of reduced OS.

When focusing on completely resected ACC (status of resection R0), an ENSAT 3 stage (*p* = 0.014), Weiss score > 6 (*p* < 0.0001), and Ki67 index > 10% (*p* < 0.0001) were associated with reduced RFS (Table 3). Strong SOAT1 expression did not, in the univariate analysis, correlate with reduced RFS (*p* = 0.24). However, when considering features of established importance in ACC prognosis, we did show that this is an important predictor of recurrence in the multivariate analysis—a Ki67 index > 10% (HR 9.87, 95% CI 4.39–22.2; *p* < 0.0001) and strong SOAT1 protein expression (HR 2.1, 95% CI 1.09–4.06; *p* = 0.027) were predictors of reduced RFS.

PFS was not evaluated through a multivariate analysis since the number of patients with metastatic disease at diagnosis was small (*n* = 22).

### 2.3. SOAT1 Protein Expression and Ki67 Index

**Figure 4:**
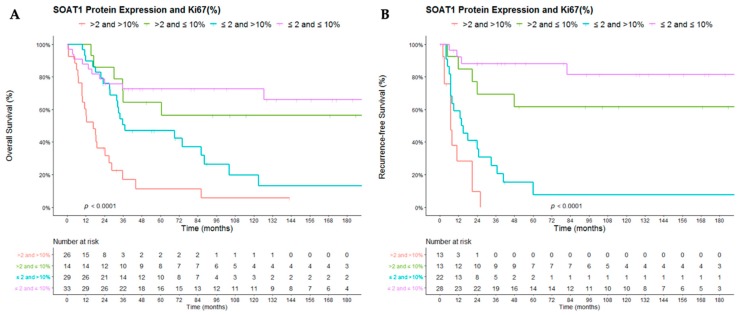
Impact of Ki67 index and SOAT1 protein expression on (**A**) overall survival and (**B**) recurrence-free survival in adult patients with ACC. For recurrence-free survival analysis, only patients with complete resection (status of resection R0) have been analyzed.

Since the Ki67 proliferation index is the most widely used prognostic factor for ACC in clinical practice, we evaluated the impact of the combination of the Ki67 index and SOAT protein expression on OS and RFS (Ki67 index available for 102 and 76 patients, respectively). The combination of a Ki67 index > 10% with strong expression of the SOAT1 protein (score > 2) significantly indicated worse outcomes compared to the presence of a Ki67 index > 10% associated with weak or absent SOAT1 expression (score ≤ 2) (HR 2.65, 95% CI 1.43–4.9; *p* = 0.002 for OS; HR 2.24, 95% CI 1.02–4.95; *p* = 0.044 for RFS), suggesting that these two prognostic markers may be complementary and may define a subgroup of patients with an even more unfavorable prognosis (Figure 4).

When the subgroup of patients with Ki67 ≤ 10% was analyzed, strong SOAT1 protein expression did not significantly impact OS (HR 1.27, CI 0.46–3.5; *p* = 0.64) and RFS (HR 2.53, 95% CI 0.68–9.43; *p* = 0.166).

### 2.4. SOAT1 Gene Expression

Similarly, high *SOAT1* gene expression was more frequent in patients with a more advanced disease stage at diagnosis (ENSAT 3 and 4, 62% (13 out of 21) versus ENSAT 1 and 2, 24%, (5 out of 21); *p* = 0.006), and in carcinomas exhibiting a higher Ki67 index (Ki67 > 10%, 76% (16 out of 21) versus Ki67 ≤ 10%, 29% (6 out of 21); *p* = 0.005). *SOAT1* gene expression did not correlate with either survival or recurrence, probably because the tumor cohort available for gene expression analysis was smaller than that for immunohistochemistry; nevertheless, the occurrence of post-translational events being responsible or contributing to this finding cannot be excluded.

## 3. Discussion

During tumorigenesis, cancer cells acquire various metabolic alterations to overcome the metabolic challenges of rapid proliferation and survival under inhospitable conditions [13]. Recent evidence has revealed that the reprogramming of lipid metabolism, including changes in de novo lipogenesis, FA oxidation, and lipid mobilization and recycling, is essential for various aspects of tumorigenesis [14]. The impact of SOAT1 overexpression on cancer prognosis has already been demonstrated in several malignancies, suggesting a pivotal role of lipid metabolism and lipid droplet formation in tumor progression and aggressiveness [15].

SOAT1 expression in the adrenal cortex is very high, as cholesterol pools in LDs protect cells against the toxic effects caused by free cholesterol but at the same time, can be rapidly released by the action of sensitive hormone lipase after stimulation of adrenocorticotropic hormone (ACTH) stimulation for steroidogenesis [5]. In ACC, the overexpression of SOAT1 would result in an even better ability to convert excess free cholesterol into cholesterol esters that are stored in LDs, thus protecting cancer cells from cholesterol accumulation and ER stress initiation and enhancing cell viability and steroidogenesis capacity through less feedback inhibition on pathways involved in steroid synthesis and tumor growth.

In 2015, Sbiera et al. demonstrated that SOAT1 protein expression in ACCs was more variable and heterogeneous than that in normal adrenal glands and adrenocortical adenomas [7]. However, the correlation of SOAT1 protein expression with ACC features and with clinical outcomes was not investigated in detail in that study.

To the best of the authors’ knowledge, this is the first study to explore the correlation between SOAT1 expression and clinical, biochemical, and histopathological features of ACC and, mainly, with the clinical outcomes of recurrence, progression, and survival. Our findings are pioneering in demonstrating that SOAT1 expression was higher in ACCs harboring features of high aggressiveness and that strong SOAT1 protein expression correlated independently with reduced RFS and OS.

The importance of the Ki67 proliferation index in the prognosis of patients with ACC is well established and unquestionable [16]. In this study, we were able to demonstrate that SOAT1 protein expression is a Ki67-independent prognostic factor and therefore may be useful in refining the risk for recurrence and death. This association is confirmed by our finding of shorter OS and RFS in patients with both Ki67 > 10% and strong expression of SOAT1. In our cohort, 18% of the patients with ACCs presenting a Ki67 index ≤ 10% still developed recurrence after complete resection of the primary tumor. In 2015, Beuschlein et al. also demonstrated that a portion of ACC patients who presented tumors with a Ki67 index < 10% evolved to have recurrence and death [16]. An analysis of the impact of SOAT1 protein expression among tumors exhibiting a Ki67 index ≤ 10% was also performed in this study; however, SOAT1 protein expression did not significantly impact OS and RFS in this subgroup. This suggests that the development of recurrence and death in these patients were not due to the strong expression of SOAT1.

The strengths of this study were the large cohort size in the context of a rare disease, the long follow-up, and the comprehensive follow-up data available for all patients. In addition, the use of tissue microarray technology, which favored uniformity of the experimental conditions, and the use of immunohistochemistry techniques, which have short execution times and low costs and are widely available, are also strengths of our study. Nonetheless, the present study has a few limitations that should be considered: this is a retrospective study, and the patients included in this study are from a single center. Heterogeneity of SOAT1 expression within a single tumor may be underestimated by using tissue microarray. Finally, response to specific treatments was not evaluated in this study. Despite the large cohort size for immunohistochemistry, the small number of cases available for gene expression analysis did not permit the correlation of *SOAT1* gene overexpression with reduced OS and RFS.

The results found here along with the results found by important previous studies raise some interesting questions to be evaluated by further studies. We did not assess the impact of SOAT1 protein expression on response to mitotane treatment. A multicentric study of patients treated with mitotane monotherapy is required and underway to answer this question. Another interesting question to be evaluated is the comparison between SOAT1 expression and SF-1 expression, considering that *SOAT1* is a target gene of SF-1 transcription factor and SF-1 expression also has prognostic value in ACC [17].

The discovery of SOAT1 protein expression as a new prognostic biomarker of ACC greatly aids professionals treating ACC in clinical practice. Since well-established treatment alternatives are limited both in terms of availability and efficacy, a refined prediction of the patients’ risk for recurrence, progression, or death is very important. A marker representing a therapeutic target is even more promising, as it allows for further efforts regarding targeted treatment strategies for this dismal malignancy.

In a previous ACC Symposium (Fifth International Adrenocortical Carcinoma Symposium, Ann Harbor, USA, 2015), data showing the efficacy of ATR-101, an orally available SOAT1 inhibitor, in preclinical models of ACC were presented. A phase I clinical trial for the safety and tolerability of ATR-101 was recently completed, but its results have not yet been published (https://clinicaltrials.gov/ct2/show/NCT01898715). According to our results, we suggest to further validate SOAT1 as a prognostic biomarker and to revisit SOAT1 as a possible therapeutic target in ACC. 

## 4. Materials and Methods

One hundred and twelve tissue samples were collected between 1979 and 2017. The samples comprised 112 ACCs from 112 independent patients who had been followed or are being followed in a unique tertiary center (Faculty of Medicine from University of São Paulo (Table 4)). All patients donating adrenocortical carcinoma tissue gave written informed consent for tissue and clinical data collection, and the study was approved by the ethics committee of the University of São Paulo (Approval code: 73921517.0.0000.0068; Date: 22 September 2017). Detailed clinical data, including clinical and hormonal presentation, and follow-up and survival data, in addition to histopathological data, were collected for all patients.

### 4.1. Tissue Microarray and Immunohistochemical Analysis

Representative areas of the ACCs (viable tumor tissue without necrosis) were identified on hematoxylin- and eosin-stained slides and marked on paraffin donor blocks. The spotted areas of the donor blocks were punched (1.0 mm punch) and mounted into three paraffin blocks using a precision microarray instrument (Beecher Instruments, Sun Prairie, WI, USA), with a total of 3 cores per ACC sample/patient. One slide from each of the three TMA paraffin blocks of the triplicate set was used for staining with anti-SOAT1 rabbit polyclonal antibody (titer 1:4000; ab39327; Abcam; Cambridge, MA, USA). An immunoperoxidase immunohistochemical modified method with humid heat antigen retrieval was used, as previously described [18]. The SOAT1 immunostaining was blindly evaluated by two independent observers (I.C.S. and M.C.N.Z.), and the mode of the two evaluations was taken for statistical analysis. The interobserver agreement coefficient (kappa) for the SOAT1 staining evaluation was 0.779 (*p* < 0.0001). A kappa coefficient > 0.6 is considered substantial agreement.

TMA samples were included in the analysis only if two or more evaluable cores were available after the staining procedure. Cytoplasmic staining was evaluated according to intensity as negative (0), low (0.5), or strong (1). The percentage of positive tumor cells was visually scored as follows: 0 if 0% of tumor cells were positive; 1 if 1%–25%, 2 if 26%–50%, 3 if 51%–75%, and 4 if 76%–100%. A semiquantitative score was then calculated by multiplying the staining intensity by the percentage of positively stained tumor cells, and the final score ranged from 0 to 4. A cut-off of 2 discriminated absent/weak staining from strong staining, a score >2 defined tumors with high SOAT1 staining, and a score ≤2 defined tumors with absent or low SOAT1 staining.

Nuclear staining for Ki67 in our cohort was also evaluated. Another set of three slides was stained with mouse monoclonal anti-human Ki67 antigen (titer 1:40; clone MIB-1; code M7240; Dako, Denmark). The stained slides were scanned by the Scanner of histological slides through the Pannoramic Viewer 1:15 software (3DHISTECH, Budapest, Hungary). All the images were processed with the Image Pro Plus 4.5 software (MediaCybernetics, Rockville, MD, USA), and the positive and total nuclei were automatically counted.

### 4.2. Quantitative Real-Time PCR (qRT-PCR)

After surgical resection of the primary tumor, tumor fragments were immediately frozen in liquid nitrogen and stored at −80 °C until total RNA extraction using the AllPrep DNA/RNA Minikit (Qiagen, Hilden, Germany). RNA samples from 42 tumors from 42 independent patients were treated with DNAse using standard procedures. Complementary DNA was generated from 1 μg of total RNA using the commercial Superscript III Reverse Transcriptase kit (Invitrogen, Carlsbad, CA, USA). Quantitative real-time polymerase chain reaction was performed in the AriaMx (Agilent; Santa Clara, CA, USA) using TaqMan gene expression assays according to the manufacturer’s instructions (Applied Biosystems; Carlsbad; CA, USA). The PCR cycling conditions were as follows: 10 min at 95 °C, 40 cycles of 95 °C for 15 s, and 60 °C for 1 min. The assay for the target gene was SOAT1 (Hs00922322_m1). β-glucuronidase (β-GUS, Hs 99999908_m1) and cyclophilin A (CYC-A, Hu_4326316E) were used as endogenous genes for normalization.

### 4.3. Statistical Analysis

Fisher’s exact test or chi-square test was applied to assess possible associations between qualitative variables. Comparisons of quantitative variables in relation to two independent groups were performed by the nonparametric Mann–Whitney U test or, when there was evidence of a normal distribution, by the *t*-test for unpaired samples (the Shapiro–Wilk test was applied to assess the normality of the data).

OS was defined as the time between the date of ACC diagnosis and the date of disease-related death or last follow-up visit. RFS was defined as the time between the date of complete tumor resection and the date of the first radiological evidence of local or distant recurrence. PFS was defined as the time between the date of diagnosis and the date of the first radiological evidence of disease progression [according to the response evaluation criteria in solid tumors (RECIST) [19]. Regarding the analysis in which the time to death, recurrence, and progression were of interest, the survival analysis technique was employed. This consisted of determining a cut-off point that best discriminated the survival curves. The cut-off values were estimated by maximizing the standardized log-rank statistic. Once the cut-off was estimated, the survival function was estimated using the Kaplan–Meier estimator, and the estimated survival curves were compared using the log-rank test. In addition, a Cox proportional hazards model was fitted to the data to describe the relationship between independent variables and time to death, recurrence, and progression. Among the variables with *p*-values less than 0.05 in the simple Cox regression model and the variables with substantial clinical importance, variables were selected for multiple Cox regression. This selection was made with a stepwise backward method with an input *p*-value of 0.25 to obtain the final model. In all adjusted models, the proportionality assumption was evaluated by Schoenfeld residuals.

The significance level adopted was 5%; that is, the results with *p*-values less than 0.05 were considered significant. Data analysis was performed using R software, version 3.5, and Statistical Package for Social Sciences (SPSS) software, version 24.

## 5. Conclusions

In conclusion, SOAT1 overexpression was an independent and significant risk factor for poor prognosis in patients with ACC in our cohort. Strong SOAT1 protein expression correlated with known features of high aggressiveness in ACC, was significantly associated with reduced overall survival and recurrence-free survival, and was an independent predictor of death and recurrence in ACC patients. These findings demonstrated that SOAT1 protein expression is a marker with prognostic value for ACC.

As SOAT1 is a drug-targetable protein, our findings reinforce the importance of investigating SOAT1 as a therapeutic target in ACC. Ultimately, these results provide a powerful tool for identifying patients with ACC with poor prognosis, and who might benefit from further targeted treatment. Validation of the results found in this study in other cohorts and using data from publicly available sources, such as The Cancer Genome Atlas, are necessary to confirm the results found in this cohort and should be the subject of subsequent studies involving this theme.

## Figures and Tables

**Table 1:** Frequency of immunoreactivity scores (0–4) for Sterol-O-acyl transferase 1 (SOAT1) protein in 112 adrenocortical carcinomas and categorization of cases according to protein expression.

Cases	SOAT1 Immunoreactivity Score						
	Weak or Absent Protein Expression (Score ≤ 2)					Strong Protein Expression (Score > 2)	
	0	0.5	1	1.5	2	3	4
*n* (%)	26 (23.2)	4 (3.6)	4 (3.6)	4 (3.6)	32 (28.5)	5 (4.5)	37 (33)
*n* (%)	70 (62.5)					42 (37.5)	

**Table 2:** Relationship between SOAT1 protein expression and baseline clinical and biochemical parameters, and surgical specimen histological characteristics from 112 adult patients with ACC (only tumor samples derived from primary surgery).

Characteristic	SOAT1 Protein Expression		*p* *
	Weak or Absent (≤2)	Strong (>2)	
*n* (%)	70 (62.5)	42 (37.5)	
Sex—*n* (%)			
Female	53 (75.7)	26 (61.9)	0.181
Male	17 (24.3)	16 (38.1)	
Age (years)			
Median	43.6 (15.38–83.19)	36.32 (17.71–81)	0.32 **
Tumor size (cm)			
Median	11.15 (2.5–27)	12.2 (2.2–23)	0.792 **
Hormonal status—*n* (%)			
Cushing	40 (58)	35 (83.3)	**0.01**
Non-Cushing	29 (42)	7 (16.7)	
Stage (ENSAT)—*n* (%)			
1–2	45 (64.3)	15 (35.7)	**0.011**
3–4	25 (35.7)	27 (64.3)	
Weiss score			
3–6	42 (62.7)	18 (42.9)	0.068
>6	25 (37.3)	24 (57.1)	
Ki67 index (%)			
Median	10 (1–75)	17.5 (1–80)	0.002 **

ENSAT, European Network for the Study of Adrenal Tumors. * Independence test (chi-square test with continuity correction or chi-square test). ** Student’s *t*-Test for independent samples.

**Table 3:** Prognostic factors for overall survival, recurrence-free survival, and progression-free survival in adult patients with ACC (only tumor samples derived from primary surgery).

Variables	Univariate Analysis			Multivariate Analysis		
	**Overall Survival**					
	HR	95% CI	*p*	HR	95% IC	*p*
Cushing’s syndrome	2.019	1.16–3.5	**0.013**			
ENSAT 3/4 stage	3.48	2.11–5.75	**<0.0001**	2.93	1.68–5.13	**<0.0001**
Weiss Score > 6	3.47	2.08–5.77	**<0.0001**			
Ki67 > 10%	3.67	2.04–6.59	**<0.0001**	3.11	1.71–5.67	**<0.0001**
Strong SOAT1 protein expression	1.94	1.19—3.15	**0.007**	2.15	1.26–3.66	**0.005**
	**Recurrence-Free Survival**					
	HR	95% CI	*p*	HR	95% IC	*p*
Cushing’s syndrome	1.16	0.64–2.11	0.619			
ENSAT 3 stage	14	1.85–105.5	**0.014**			
Weiss Score > 6	3.33	1.8–6.15	**<0.0001**			
Ki67 > 10%	9.05	4.12–19.81	**<0.0001**	9.87	4.39–22.2	**<0.0001**
Strong SOAT1 protein expression	1.44	0.78–2.67	0.241	2.1	1.09–4.06	**0.027**
	**Progression-Free Survival**					
	HR	95% CI	*p*	HR	95% IC	*p*
Cushing’s syndrome	1.64	0.37–7.26	0.514			
Weiss Score > 6	2.45	0.52–11.43	0.252			
Ki67 > 10%	3.37	0.44–25.64	0.24			
Strong SOAT1 protein expression	4.1	1.14–14.7	**0.03**			

HR, hazard ratio; CI, confidence interval; ENSAT, European Network for the Study of Adrenal Tumors.

**Table 4:** Clinical and tumor characteristics of 112 adult patients with ACC included in the study.

Characteristic	Adrenocortical Carcinomas *n* = 112
Age (years) ^(A)^	40 (15–83)
Sex (Female:Male)	2.4:1
Clinical syndrome, *n* (%)	
Cushing syndrome	26 (23)
Virilizing syndrome	10 (9)
Cushing and virilizing syndrome	51 (46)
Non-functioning	20 (18)
Not available	5 (4)
ENSAT, *n* (%)	
1	10 (9)
2	50 (45)
3	27 (24)
4	25 (22)
Weiss score, *n* (%)	
3–6	63 (56)
>6	49 (44)
Ki67, *n* (%)	
Known	102 (91)
≤10%	47 (46)
>10%	55 (54)
Follow-up (months) ^(A)^	35 (0.6–381)

A: median (range).
